# Triglyceride-Glucose Index and Cardiovascular Events in Kidney Transplant Recipients

**DOI:** 10.1016/j.ekir.2023.08.021

**Published:** 2023-08-23

**Authors:** Mathilde Colladant, Melchior Chabannes, Thomas Crepin, Jamal Bamoulid, Cécile Courivaud, Didier Ducloux

**Affiliations:** 1 Univ. Franche-Comté, INSERM, Etablissement Français du Sang Bourgogne Franche-Comté, UMR1098, RIGHT Interactions Hôte-Greffon-Tumeur/Ingénierie Cellulaire et Génique, Besançon, France; 2 Department of Nephrology, Besançon University Hospital, Besançon, France

**Keywords:** cardiovascular disease, kidney transplantation, metabolic syndrome, triglyceride glucose index

## Abstract

**Introduction:**

Kidney transplant recipients (KTRs) have an increased risk of cardiovascular (CV) events (CVEs) compared with the general population. The impact of insulin resistance on CV risk after transplantation is not well defined.

**Methods:**

We tested whether triglyceride-glucose (TyG) index, a surrogate marker of insulin resistance, may predict posttransplant CVEs in a cohort of 715 consecutive KTRs all included 1 year after transplant.

**Results:**

Follow-up was 9.1 ± 4.6 years. Mean TyG at inclusion was 4.75 ± 0.29 (median, 4.73 [4.14–5.84]). In multiple regression analysis, having a TyG above the median value was associated with higher body mass index (BMI), low high-density lipoprotein (HDL) cholesterol level, and greater urinary protein excretion. A total of 127 CVEs (17.7%) occurred during the study period. In univariate analysis, TyG was strongly associated with CVE occurrence (hazard ratio [HR] 2.06, 95% CI 1.42–3.50, for each increase of 0.1 in TyG, *P* < 0.001). The best predictive value was 4.87 (HR 6.32, 95% CI 3.30–12.11, *P* < 0.001). The risk of CVE gradually increased with higher TyG index (quartile 2, HR 1.71, 95% CI 0.84–5.20, *P* = 0.139; quartile 3, HR 3.12, 95% CI 1.61–6.02, *P* < 0.001; quartile 4, HR 7.46, 95% CI 4.03–13.80, *P* < 0.001, vs. quartile 1). TyG remained associated with CVE in multivariate analysis (HR 2.11, 95% CI 1.22–3.68, for each increase of 0.1 in TyG, *P* < 0.001).

**Conclusion:**

Insulin resistance, as measured by the TyG index is strongly associated with CVE in KTRs. Improving insulin sensitivity seems to be a major issue to prevent CV morbidity and mortality in this high-risk population.

## Graphical abstract

**Figure:**
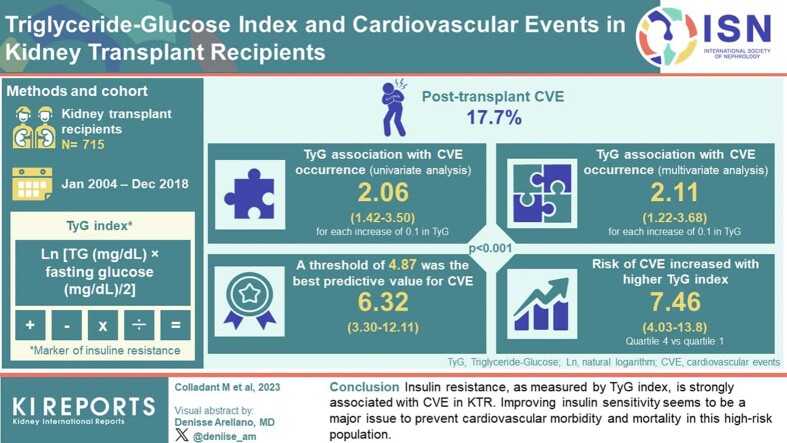

KTRs have an increased risk of CVEs compared with the general population.^1^ This is, in great part, linked to a very high frequency of traditional CV risk factors.^2^^,^^3^ A great number of traditional CV risk factors, such as diabetes, low HDL cholesterol, or hypertension, are related to insulin resistance. Although insulin resistance is likely to be frequent after kidney transplantation, reliable measure of frequency is lacking. Reduced renal function and immunosuppressive drugs are some factors which could participate in the high prevalence of insulin resistance in KTRs.^4^ In addition, metabolic syndrome (MS), which is closely linked to insulin resistance, is observed in one-third of KTRs 1 year posttransplant and is associated with CVE.^5^ Moreover, a large part of KTRs had either diabetes before transplantation or develop *de novo* diabetes after transplantation,^6^ and diabetes and even prediabetes are associated with the occurrence of posttransplant CVE.^7^

Nevertheless, although all these examples suggest the role of insulin resistance, they relate to more than only insulin resistance. Indeed, MS also depends on chronic low-grade inflammation which indisputably contributes to CVD in KTRs.^8^ However, posttransplant diabetes is mainly related to beta cell dysfunction rather than insulin resistance.^9^^,^^10^

TyG index is a simple, reliable, and objective surrogate marker of insulin resistance, and some studies suggest that it is a better marker of insulin resistance than Homeostatic Model Assessment for Insulin Resistance.^11^^,^^12^ In this study, we tested whether TyG index may predict posttransplant CVE in a large cohort of KTRs with a follow-up of almost 10 years.

## Methods

### Study Design and Populations

Research has been conducted in 715 consecutive patients transplanted between January 1, 2004, and December 31, 2018, in the Transplant Unit of the CHU of Besancon and having at least 1 year follow-up after the transplant. A total of 65 patients who died or lost their graft in the first-year posttransplant had been excluded.

Among these 715 KTRs, 221 patients (31%) had received T cell-depleting antithymocyte globulin therapy and 494 (69%) had received nondepleting α-CD25 monoclonal antibody therapy. Tacrolimus and mycophenolate mofetil were used as first immunosuppressive regimen in all patients. Targeted trough levels of tacrolimus were between 6 and 8 ng/ml during the first year and between 4 and 6 ng/ml after (except in cases of acute rejection in the first-year posttransplant or for those having developed donor-specific antibodies). Steroids were withdrawn between the third- and the sixth-month posttransplant (except for those who underwent acute rejection before 3 months). All the transplants were performed with a negative crossmatch.

Cytomegalovirus prophylaxis was given according to a standardized protocol. All cytomegalovirus-exposed patients received valganciclovir for 3 months. All cytomegalovirus-naive patients having received a cytomegalovirus-positive kidney received valganciclovir for 3 or 6 months. All patients received *Pneumocystis* antimicrobial prophylaxis with trimethoprim-sulfamethoxazole for at least 6 months.

Database was closed for analysis on December 31, 2021. Minimum follow-up was 3-year posttransplant.

### TyG Index

The TyG index was calculated by using the following formula: Ln (TG [mg/dl] × fasting glucose [mg/dl]/2).^13^

### Confounding Factors

Age, sex, BMI, abdominal circumference, diabetes, hypertension (treatment with antihypertensive drugs), statin use, smoking habit, immunosuppressive drugs were analyzed as covariates. Concerning abdominal obesity, waist circumference was measured during dietetic consultation. Abdominal obesity was defined as a waist circumference of more than 88 cm in women and more than 102 in men. MS was present when at least 3 among the following 5 conditions were observed: waist circumference >88 cm in women or >102 cm in men; triglyceride level above 150 mg/dl; hypertension; HDL cholesterol under 50 mg/dl in women or 40 mg/dl in men; and fasting glucose level above 100 mg/dl. Dialysis mode (none, hemodialysis, or peritoneal dialysis) and its duration before transplantation were also recorded. Delayed graft function and acute rejection occurred during the first-year posttransplant. Other relevant immunologic parameters, such as pretransplant panel-reactive antibodies (0 vs. positive panel-reactive antibodies at any level), current donor-specific antibodies, and transplant rank (first vs. second or more), were analyzed as covariates. Diverse biochemical parameters were also analyzed (total cholesterol, HDL cholesterol, creatinine). Urine protein/creatinine ratio was assessed as the protein/creatinine ratio (g/g). Glomerular filtration rate was calculated with the CKD-Epidemiology Collaboration equation.^14^

LDL cholesterol was estimated through the Friedewald formula.^15^

Methods of assessment and definitions of these variables have been previously described in detail.^16^

### Outcomes

The primary end point was the effect of TyG on CV event-free survival defined as the time elapsed between 1-year post-transplant and the first CVE. Atherosclerotic events were considered as previously reported.^8^

Myocardial infarction was documented by serial 12-lead electrocardiogram evidence or Q-wave infarction and appropriate myocardial enzyme level elevations, coronary revascularization including coronary artery bypass surgery or percutaneous transluminal coronary angioplasty, or typical history of angina with abnormal coronarography result.

Both nonhemorrhagic and hemorrhagic strokes were confirmed by neurologic examination findings consistent with new-onset focal neurologic deficits, with or without computed tomography or magnetic resonance imaging evidence of cerebral infarction or symptomatic extracranial artery stenosis resulting in carotid endarterectomy.

Doppler or arteriography findings were used to confirm the presence of abdominal aortic repair, lower extremity revascularization through bypass surgery or angioplasty, lower extremity amputation, or new onset of intermittent claudication. Two physicians independent of the study were responsible for diagnostic ascertainment. This analysis was performed without knowledge of baseline characteristics. CVEs were retrospectively recorded.

### Statistical Analysis

Median (interquartile range), mean values (SD), and frequency (percentage) were provided for continuous and categorical variables, respectively. Medians, means, and proportions were compared using Student *t*-test and chi-square test (or Fisher exact test, if appropriate), respectively. Correlations between variables were assessed by Pearson correlation coefficient.

Receiving operator characteristic curve was used to determine different diagnostic abilities.

In some analyses, age (<53 and ≥53 years) was split in 2 classes, as were glomerular filtration rate (<52 and ≥52 ml/min) and C-reactive protein (CRP) levels (<3 and ≥3 mg/l [median values]).

Follow-up duration was calculated using a reverse Kaplan-Meier estimation.

Cox proportional hazard models were performed to estimate HR and 95% CI for factors associated with CVE. The association of baseline parameters with the occurrence of a CVE was first assessed using univariate Cox analyses, and then parameters with *P* values < 0.05 were entered into a final multivariable Cox regression model. The assumption of proportionality was checked by plotting log-minus-log survival curves and by cumulative martingale process plots.

## Results

### Characteristics of the Study Population

Characteristics of the study population are depicted in Tables 1 and 2. Mean follow-up was 9.1 ± 4.6 years.

**Table 1:** Characteristics of the study population

Patients' characteristics	Mean ± SD
Age (yr)	52 ± 13
Sex (% male)	64%
First transplant (%)	89%
PRA (%>0)	15%
DGF	21%
Acute rejection	10%
Diabetes (%)	33%
Current smoking (%)	22%
Hypertension (%)	79%
BMI (kg/m^2^)	26 ± 5.1
Abdominal obesity (%)	39%
Statins (%)	75%
Azathioprine (%)	11%
MMF (%)	82%
Calcineurin inhibitors (%)	85%
mTOR inhibitors (%)	8%
Belatacept (%)	8%

BMI, body mass index; DGF, delyaed graft function; MMF, mycophenolate mofetil; mTOR, mammalian target of rapamycin; PRA, panel reactive antibody.

**Table 2:** Biological characteristics at the entry in the study

Patients' characteristics	Median ± SD	Median (25–75 IQR)
Glucose (mg/dl)	108 ± 38	95 (86–116)
Triglycerides (mg/dl)	144 ± 76	126 (91–178)
TyG	4.75 ± 0.3	4.73 (4.53–4.93)
HDL cholesterol (mg/dl)	17.8 ± 3.9	17.4 (13.5–20.5)
LDL cholesterol (mg/dl)	31.7 ± 1.6	31.2 (23.2–37.1)
Uric acid (mmol/l)	416 ± 102	408 (338–482)
Serum creatinine (μmol/l)	130 ± 45	121 (101–153)
eGFR (ml/min per 1.73 m^2^)	54 ± 19	52 (41–66)
UPCR (g/g of creatinine)	0.40 ± 0.51	0.27 (0.1–0.47)

eGFR, estimated glomerular filtration rate; HDL, high-density lipoprotein; IQR, interquartile; LDL, low-density lipoprotein; TyG, triglyceride-glucose; UPCR, urine protein/creatinine ratio.

### TyG Index

Mean TyG was 4.75 ± 0.29 (median, 4.73 [4.14–5.84]). Distribution of values was normal.

TyG was highly related to factors associated with MS: HDL (r = −0.33, *P* < 0.001), BMI (r = 0.38, *P* < 0.001), and uric acid (r = 0.24, *P* < 0.001). TyG was higher in patients with diabetes (4.92 ± 0.29 vs. 4.67 ± 0.24, *P* < 0.001) and in those with hypertension (4.77 ± 0.29 vs. 4.66 ± 0.25, *P* = 0.009). Patients with abnormal abdominal perimeter had higher TyG (4.89 ± 0.29 vs. 4.67 ± 0.24, *P* < 0.001).

There were 329 patients (46%) who met the criteria for MS. TyG index was higher in patients with MS (4.94 ± 0.25 vs. 4.59 ± 0.20, *P* < 0.001). Area under the curve of receiving operator characteristic curve was 0.88 (0.86–0.91) (*P* < 0.001). A TyG index of 4.74 was the best predictive value with a sensitivity of 84% and a specificity of 82%.

TyG correlates with age (r = 0.24, *P* < 0.001). Worse renal function (r = −0.30, *P* < 0.001) and higher urine protein/creatinine ratio (r = 0.32, *P* < 0.001) were also related to TyG.

TyG was not associated with any immunosuppressive drug. TyG was similar in patients receiving statins and in those not receiving statins (4.73 ± 0.30 vs. 4.75 ± 0.28, *P* = 0.611).

In multiple regression analysis, having a TyG above the median value was associated with higher BMI (odds ratio 1.22 [1.12–1.31 for each increase of 1 kg/m^2^], *P* < 0.001), low HDL cholesterol (odds ratio 0.6 [0.06–0.94] for each increase of 0.1 g/l, *P* = 0.023), and greater urine protein/creatinine ratio (odds ratio 3.46 [1.47–8.16] for each increase of 1 g/g of creatinine, *P* = 0.005). These 3 factors explain 79% of the variation in TyG.

### CVEs

**Figure 1:**
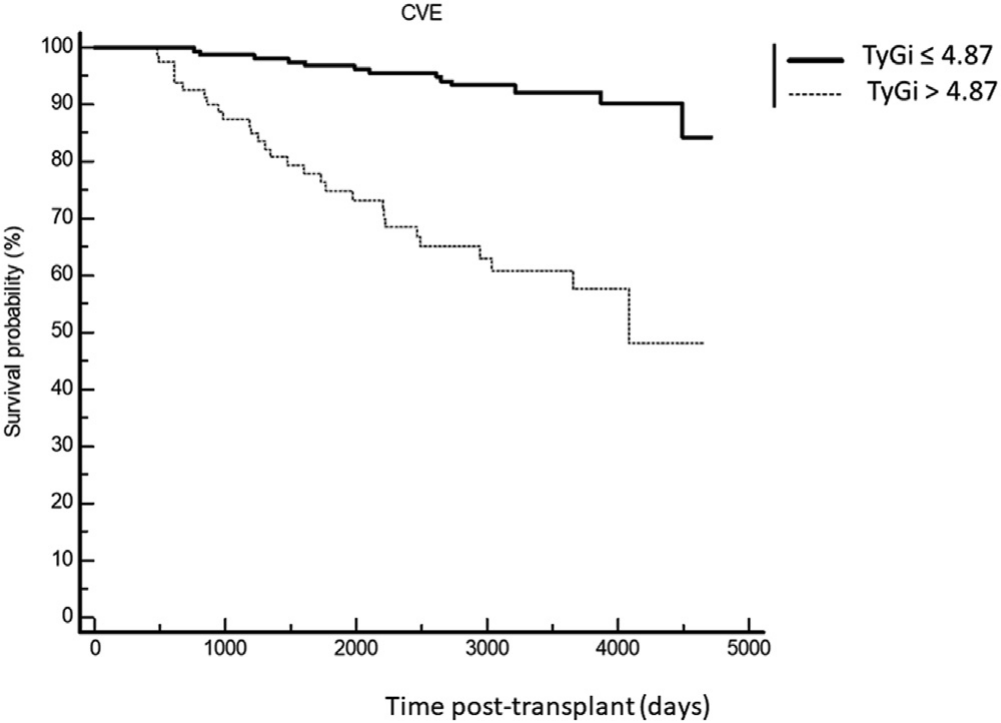
Kaplan-Meier survival curves comparing patients with TyG index ≤ 4.87 or >4.87. CVE, cardiovascular event; TyG, triglyceride-glucose; TyGi, TyG index.

A total of 127 CVEs (17.7%) occurred during the study period. TyG was strongly associated with CVE occurrence (HR 2.06, 95% CI 1.42–3.50, for each increase of 0.1 in TyG, *P* < 0.001). The best predictive value was 4.87 (HR 6.32, 95% CI 3.30–12.11, *P* < 0.001) (Figure 1).

The risk of CVE gradually increased with higher TyG index (quartile 2, HR 1.71, 95% CI 0.84–5.20, *P* = 0.139; quartile 3, HR 3.12, 95% CI 1.61–6.02, *P* < 0.001; quartile 4, HR 7.46, 95% CI 4.03–13.80, *P* < 0.001; vs. quartile 1).

Because TyG was associated with BMI, HDL levels, and urine protein/creatinine ratio, we first performed bivariate analyses to assess whether TyG was still associated with CVE after adjustment for these confounding variables. TyG remained associated with CVE after these different adjustments.

Triglycerides (HR 1.06, 95% CI 1.04–1.09, for each increase of 10 mg/dl, *P* < 0.001) and plasma glucose level (HR 1.10, 95% CI 1.06–1.14, for each increase of 10 mg/dl, *P* < 0.001) were also associated with CVE. Nevertheless, after adjustment for both triglycerides and glucose level, only TyG remained associated with CVE (HR 2.55, 95% CI 1.84–3.76, for each increase of 0.1 in TyG, *P* < 0.001).

Even after adjustment for MS, TyG index remained associated with CVE (HR 1.84, 95% CI 1.25–3.45, for each increase of 0.1 in TyG, *P* < 0.001). The incidence of CVE in patients with MS but with TyG index under the median value was 7.8% whereas it was 15.6% and 29.5% in patients with TyG index value above the median without and with MS, respectively.

Subanalysis in different subcategories of patients confirmed the association between TyG and CVE even when this association seems to be lesser in patients with diabetes (Table 3).

**Table 3:** Association between TyG and cardiovascular events in different patients’ categories

Patients' characteristics	Subgroup	HR	95% CI	*P*-value
Age	<53	1.44	1.09–2.53	0.002
	≥53	1.30	1.06–1.87	0.003
Sex	Female	1.53	1.06–3.37	0.011
	Male	1.46	1.13–1.88	<0.001
Diabetes	No	1.93	1.19–3.56	<0.001
	Yes	1.23	1.01–1.64	0.044
eGFR	<52	1.17	1.03–1.48	0.011
	≥52	1.69	1.03–3.47	0.025

eGFR, estimated glomerular filtration rate; HR, hazard ratio; TyG, triglyceride-glucose.

Age (HR 1.06, 95% CI 1.03–1.09, for each supplementary year, *P* < 0.001), sex (HR 3.26, 95% CI 2.05–5.20, *P* = 0.005), smoking status (HR 1.98, 95% CI 1.55–2.62, *P* < 0.001), diabetes (HR 2.34, 95% CI 1.29–4.26, *P* = 0.005), a past history of CVE (HR 2.47, 95% CI 1.34–6.42, *P* < 0.001), uric acid (HR 1.04, 95% CI 1.02–1.07, for each increase of 10 μmol/l, *P* < 0.001), higher CRP (>3 mg/dl, median value) (HR 3.22, 95% CI 1.17–5.67, *P* = 0.005), glomerular filtration rate (HR 0.97, 95% CI 0.95–0.99, for each increase of 1 ml/min per 1.73 m^2^, *P* = 0.001), and urinary protein excretion (HR 1.35, 95% CI 1.09–1.69, for each increase of 1 g/g, *P* = 0.001) were also associated with CVE (Table 4).

**Table 4:** Factors associated with cardiovascular events

Patients' characteristics	Univariate			Multivariate		
	HR	95% CI	*P-*value	HR	95% CI	*P*-value
Age^a^	1.06	1.03–1.09	<0.001	1.04	1.03–1.06	<0.001
Sex^b^	3.26	2.05–5.20	<0.001	2.62	1.60–4.31	0.021
Diabetes	2.34	1.29–4.26	0.005	1.28	0.81–2.04	0.283
Smoking	1.98	1.55–2.62	<0.001	1.70	1.31–2.32	<0.001
BMI^c^	1.03	1.01–1.0**7**	0.021	0.94	0.84–1.03	0.089
AO	1.92	1.36–2.72	<0.001	0.89	0.49–1.62	0.698
Previous history of CVE	2.47	1.34–6.42	<0.001	2.04	1.19–3.50	<0.001
Hypertension	1.55	0.95–2.56	0.081	0.92	0.52–1.62	0.776
TyG^d^	2.16	1.63–2.85	<0.001	2.14	1.27–3.84	<0.001
HDL^d^	0.41	0.08–0.81	<0.001	1.33	0.27–6.68	0.729
LDL^d^	1.44	0.76–2.71	0.268			
Uric acid^e^	1.04	1.02–1.07	0.005	1.02	0.98–1.04	0.159
CRP (>3 mg/dl)	3.22	1.17–5.67	0.005	1.98	1.07–4.32	0.037
eGFR^f^	0.97	0.95–0.99	<0.001	0.99	0.98–1.01	0.517
UPCR^g^	1.35	1.09–1.69	0.001	0.73	0.51–1.06	0.096

AO, abdominal obesity; BMI, body mass index; CRP, C-reactive protein; CVE, cardiovascular event; eGFR, estimated glomerular filtration rate; HDL, high-density lipoprotein; HR, hazard ratio; LDL, low-density lipoprotein; TyG, triglyceride-glucose; UPCR, urine protein/creatinine ratio.

a For 1-year increase in age.

b Male vs. female.

c For each increase of 1 kg/m^2^.

d For each increase of 10 mg/dl.

e For each increase of 10 μmol/l.

f For each increase of 1 ml/min per 1.73 m^2^.

g For each increase on 0.1 g/g of creatinine.

Multivariate analysis retained age (HR 1.04, 95% CI 1.03–1.06, for each supplementary year, *P* = 0.013), male sex (HR 2.62, 95% CI 1.60–4.31, *P* = 0.023), a past history of CVE (HR 2.04, 95% CI 1.19–3.50, *P* < 0.001), smoking (HR 1.70, 95% CI 1.31–2.32, *P* < 0.001), higher CRP (HR 1.98, 95% CI 1.07–4.32, *P* = 0.037), and TyG index (HR 2.14, 95% CI 1.27–3.84, for each increase of 0.1 in TyG index, *P* < 0.001) as being associated with CVE (Table 4).

## Discussion

This study cohort reports that a higher TyG index is associated with higher incidence of post-transplant CVEs. The association is independent of all other major CV risk factors. Subgroup analyses confirmed the association between TyG index and CVE in all patients’ categories. We observed these results in a large cohort of patients with a long follow-up. Finally, diagnostic ascertainment was independent of knowledge of TyG values. This finding reveals that high TyG, a surrogate marker of insulin resistance, is a potent risk factor for CV morbidity in KTR.

Our results are consistent with those of most previous studies in other patients’ population.

The association of the TyG index with CV diseases and mortality has mainly been reported in Asia.^17^, ^18^, ^19^ However, a recent study in Italy described similar association between TyG and incident cardiac events in patients with known or suspected coronary heart disease.^20^ Recently, Lopez-Jaramillo *et al.*^21^ reported on the association of TyG index with mortality and CV diseases in individuals from 5 continents. They observed that higher levels of TyG were associated with CV end points only in patients from low- or middle-income countries. This may suggest that the impact of insulin resistance on CV outcomes could be different according to income or genetic background.

However, TyG was associated with major cardiac events in all studies including patients with chronic kidney disease.^22^^,^^23^ Moreover, previous studies, using different surrogate markers of insulin resistance, have been associated with the occurrence of CVE in patients with CKD.^24^ Altogether, these results and ours suggest that physicians should have a special attention to insulin resistance as a CV risk factor.

A major issue for the clinical use of a marker is to determine a threshold. A threshold of 4.87 had the best predictive value for CVE occurrence and included 35% of the study population.

In the general population, a TyG value above 4.49 was found to be the best cutoff to define insulin resistance.^25^ In this cohort, almost 80% of patients had a TyG index above 4.49 illustrating the frequency of insulin resistance. This may partly explain the high incidence of CVEs in KTRs. Insulin resistance contributes to CV damages in many ways, some direct and others indirect. Insulin is crucial to maintain vascular integrity by supporting nitric oxide synthesis in endothelial cells and inhibiting growth and migration of vascular smooth muscle cell.^26^ Moreover, hyperinsulinemia increases the production and release of endothelin-1 by endothelial cells.^26^ Consequently, insulin resistance is associated with an imbalance between nitric oxide and endothelin-1 resulting in vasoconstriction and proliferation of vascular smooth muscle cells. Insulin resistance is also associated with low-grade inflammation. Indeed, white adipose tissue produces several adipocytokines with inflammatory properties such as interleukin-6, tumor necrosis factor-α, and monocyte chemoattractant protein-1 among others.^27^^,^^28^ Thus, we previously revealed that interleukin-6 levels were higher and that interleukin-6 production capacity was associated with incident CVEs in obese KTRs.^29^ In parallel, insulin resistance is associated with low HDL cholesterol and hypertension that may contribute to increase CV risk.^30^ Consequently, insulin resistance is *per se* responsible for vascular dysfunction and is associated with pro-atherogenic conditions.

Although the pathogenesis of MS has not been clearly elucidated, insulin resistance and chronic low-grade inflammation derived from central obesity are the most widely accepted as underlying pathophysiology. We previously reported that MS was a strong risk factor for incident CVEs in KTRs.^5^ TyG is also a strong surrogate marker of MS in this population. However, TyG index is better associated with CVE than MS itself. Indeed, increasing levels of TyG are also predictive of CVE in patients without MS. Patients with MS exhibit low-grade inflammation as measured by CRP. We observed a correlation between TyG index and CRP. However, TyG predicted CVE independently of inflammation.

Except smoking, TyG index was the only modifiable factor associated with CVE in this transplant cohort with most patients already under statins. As TyG index is mainly a surrogate marker of insulin resistance, interventions aiming to enhance insulin sensitivity should be encouraged and tested in future trials. In this strategy, weight reduction and increase in exercise are crucial. Regular exercise and dietary interventions are associated with weight loss and reduction of fat mass in KTRs.^31^^,^^32^ However, in these studies, no clear effects were observed on glucose metabolism. Nevertheless, in patients with early type 2 diabetes, intensive lifestyle intervention allows significant weight loss and is associated with diabetes remission in approximately 50% of patients.^33^^,^^34^ However, these measures seem to have to be very intense to be effective. Of note, both vegetable intake and Mediterranean diet style are associated with a decreased risk of post-transplantation diabetes, suggesting a favorable effect on insulin sensitivity.^35^^,^^36^ Some drugs act as improving insulin sensitivity. However, their use is restrained to patients with diabetes. Hypertriglyceridemia could be a therapeutic strategy. The REDUCE-IT trial^37^ revealed that the addition of icosapent ethyl, a highly purified form of eicosapentaenoic acid, reduced ischemic events among statin-treated individuals with elevated triglyceride levels and high CV risk. Nevertheless, in this study, greater TG lowering was not predictive of better CV prevention. Studies testing niacin or omega-3 to reduce CVE failed although significant decrease in TG was observed.^38^^,^^39^ Finally, a large meta-analysis revealed that fibrates could be moderately effective in secondary prevention.^40^ Altogether, these results suggest that although TG and TyG are potential biomarker of CV risk, there are few evidences that TG lowering by itself is an effective strategy for reducing such risk.

A large part of the patients was under statins (75%). Those patients were more prone to be older and to have diabetes and/or a previous history of CVE. An indication bias is consequently obvious making the association between LDL cholesterol and CVE difficult to assess. Nevertheless, we observed a strong positive association between LDL cholesterol and incident CVE in patients not receiving statins (data not revealed). Furthermore, we only included patients having a functional graft 1-year posttransplant. Even when it is by the way a survival cohort, we previously reported that metabolic parameters drastically change during the first-year posttransplant making interpretation of pretransplant parameters impossible.

Insulin resistance, as measured by the TyG index, is strongly associated with CVEs in KTRs. Improving insulin sensitivity seems to be a major issue to prevent CV morbidity and mortality in this high-risk population.

## Disclosure

All authors declare no support from any organizations for the submitted work; no financial relationships with any organizations that might have in interest in the submitted work in the previous 3 years; and no other relationships or activities that could seem to have influenced the submitted work.
